# Assembling Magnetic Nanoparticles on Nanomechanical Resonators for Torque Magnetometry

**DOI:** 10.3390/ijms21030984

**Published:** 2020-02-02

**Authors:** Tayyaba Firdous, David K. Potter

**Affiliations:** 1 Department of Physics, University of Alberta, Edmonton, AB T6G 2E1, Canada; tfirdous@ualberta.ca; 2 National Research Council of Canada, 11421 Saskatchewan Dr NW, Edmonton, AB T6G 2M9, Canada

**Keywords:** self-assembly, magnetic nanoparticles, superparamagnetic, stable single domain particles, nanomechanical resonators, torque magnetometry

## Abstract

We report a highly compliant process for patterning nanoparticle arrays on micro- and nanomechanical devices. The distinctive step involves the single layer self-assembled nanoparticles on top of released nanomechanical devices. We demonstrate the process by fabricating sizable arrays of nanomechanical devices on silicon-on-insulator substrates, acting as nanomechanical torque magnetometers. Later, the nanoparticles were self-assembled in geometrical shapes on top of the devices by a unique combination of top-down and bottom-up methods. The self-assembled array of nanoparticles successfully showed a magnetic torque signal by magnetic actuation of the magnetometer. This patterning process can be generalized for any shape and for a wide range of nanoparticles on the nanomechanical resonators.

## 1. Introduction

Magnetic nanoparticles are used in biomedicine both as contrast agents and to help cure diseases (e.g., cancer using magnetic hyperthermia) [1], and in the oil and gas industry both as contrast agents and for enhanced oil recovery [2]. Nanoparticles have to be compatible with the environment in which they are used, for example they should be biocompatible, nontoxic, have low protein absorption, and have integrative capability for therapeutic and diagnostic techniques for nanomedicine applications to reduce any side effects. Iron oxide nanoparticles are one of the best candidates for their functionalization capability, biocompatibility, and theragnostic potential. Therefore, the study of their magnetic properties for such applications is essential, especially for applications that require controlled small amounts of magnetic nanoparticles. Furthermore, the fundamental study of small-scale self-assembly of nanoparticles is also of great importance.

The measurement of the magnetic properties of a small-scale assembly of nanoparticles has been a challenge. The magnetic properties of ~350 magnetite nanoparticles by nanomechanical torque magnetometry, assembled by a Nano eNabler, was presented in [3]. However, information was lost due to the random assembly of the nanoparticles on the device, and the magnetic properties averaged out along any given axis. Therefore, a geometrical order to the magnetic nanoparticle patterning is desirable to rule out the random averaging, and to get a better insight into the magnetic properties along a particular direction. This article will discuss the procedures for assembling magnetic nanoparticles into geometrical shapes, and the nanomechanical magnetic torque of the assemblies.

There are top-down and bottom-up approaches for building nanostructures in an orderly manner. Usually, the top-down and bottom-up designs are used separately to accomplish certain processes. There have been great efforts toward organizing nanostructures on silicon surfaces [4], but we demonstrate the patterning of nanoparticles on magnetometer devices for the first time. Here, we use both processes—top-down and bottom-up—to achieve the required patterning of nanoparticles on the silicon-on-insulator (SOI) devices. The top-down process includes making the devices and controlling the shape of the nanostructures, whereas the bottom-up process includes the self-assembly of nanoparticles. The SOI devices have advantages of high sensitivity and good resonance modes, along with the elimination of membrane modes as in the case of devices made in a silicon nitride membrane [3]. The difficulties in device fabrication in a silicon nitride membrane is represented in the Supplementary Materials.

Of the main magnetometry methods, superconducting quantum interference devices ( SQUIDS) and vibrating sample magnetometery (VSM) are most commonly used for characterizing magnetic nanoparticles. SQUIDs are vector magnetometers, but need low temperature to operate [5]. Vibrating sample magnetometers (VSM) are not useful for sensitive and delicate samples because of sample vibration, heat, and reduced sensitivity [6]. However, in comparison to these magnetometry methods, the nanomechanical torque magnetometry method provides direct, linear, noninvasive, sensitive, and broadband measurements (DC magnetization to high frequency AC susceptibility). It also provides the complex volumetric magnetization in vector form and fast acquisition of the magnetic hysteresis loop, while additionally being able to capture the associated RF susceptibility.

## 2. Nanomechanical Device Fabrication by a Top-Down Approach

Nanomechanical torsional resonators were first fabricated on an industrial SOI chip, following the procedure in [7], compatible with standard nanofabrication technology. First, it involves a primitive electron beam lithography (EBL) process followed by the release of nanomechanical torsional devices in a timed buffered oxide etch (BOE). Various devices were made on different chips using positive and negative resists (a resist is a material used as a mask for electron beam lithography) for generalizing the process (see details in Supplementary Materials).

Two kinds of nanoparticle assembly, detailed in sections below, were achieved by using the positive and negative resists, hydrogen silsesquioxane (HSQ) and α−methylstyrene (ZEP), respectively, in device nanofabrication. The negative resist helps etch away all the top silicon layer (300 nm thick), except the devices. This helps to assemble nanoparticles of the device shape, whereas devices made with the positive resist are good for preferred geometrical shapes of nanoparticles on the device followed by a secondary EBL step. The secondary EBL step allows the selective shapes of nanoparticle assembly. This two step EBL process for the device fabrication and nanoparticle deposition is detailed in the Supplementary Materials and in the following sections.

## 3. Nanoparticles Self-Assembly by a Bottom-Up Approach

### 3.1. “Coffee Stain" Phenomenon and Its Removal

Self-assembly, being the root of the bottom-up nanofabrication approach, allows the nanoparticles to pattern themselves in a geometrical order. It allows the spontaneous organization of the nanoparticles. The self-assembly can be direct or indirect, free, or assisted, depending upon the process involved [8]. It includes template-assisted direct self-assembly [9], template-free direct self-assembly [10,11] externally directed self-assembly [4,12] (electric [13], magnetic [14], and flow fields [15]), self-assembly at liquid interfaces [16], self-assembly by wetting-dewetting [17], scanning probe lithography [18], and nano-imprinting [19].

**Figure 1:**
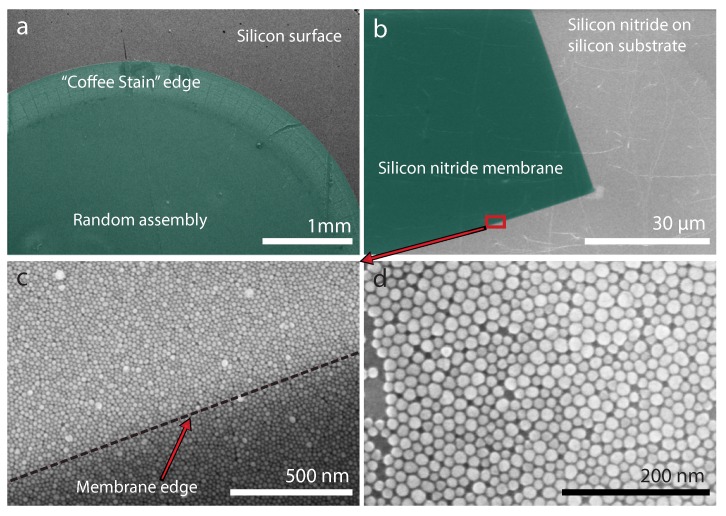
SEM images of self-assembly of nanoparticles. (**a**) Green color (false-color) represents the nanoparticle deposition on the silicon surface. (**b**) Nanoparticle self-assembly on a large scale; green color (false-color) shows the silicon nitride window (**c**) Magnified view of the edge of the silicon nitride window from (**b**) with the nanoparticle assembly (**d**) Magnified view of the close packed nanoparticle distribution in the self-assembly.

In self-assembly, fluid dynamics are involved that can describe the simple to complex nanostructure formation of multiple nanoparticles [20]. The dynamics form a “coffee stain” at initial stages when the ferrofluid (nanoparticle suspension) is dropped on top of a silicon surface as shown in Figure 1a. The “coffee stain” pattern and other formations evolve due to evaporation of the fluid. The parameters that influence the evaporation rate are the nanoparticle–liquid interactions, concentration of nanoparticles, nanoparticle and target surface chemistry, volume of droplet, suspension quality of ferrofluid and environment temperature, and pressure conditions. To achieve uniform single layer self-assembly of nanoparticles, the “coffee stain” pattern is not ideal. The experiment must be performed in an environment where the above-mentioned parameters can be controlled to reduce the “coffee stain” effect.

**Figure 2:**
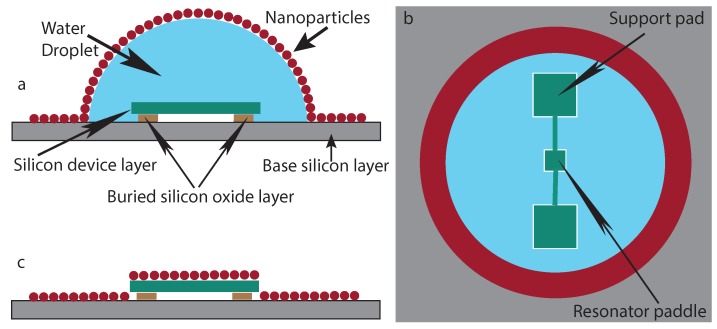
Schematic of self-assembly of nanoparticles (not to scale). (**a**) Side view schematic of nanoparticle deposition. Gray color represents the base silicon (700 μm thick), brown color is the silicon oxide layer (1 μm thick), and green color is the top silicon layer used as the device layer (300 nm thick). Water is displayed in blue while the nanoparticles are represented in red. (**b**) Top view of the schematic. The nanoparticles actually cover the water droplet and the device but are omitted in part of this view to show the device shape. (**c**) Side view of the device and nanoparticles after the drying procedure.

To minimize the “coffee stain” effect [21], self-assembly at a liquid interface [16], in a controlled environment to tune the above-mentioned parameters, was chosen to get a single layer of self-assembled nanoparticles on the device surface. A general procedure employed here involves a deionized water droplet placed on a clean surface, and then the nanoparticles are dropped on top of the water droplet. The additional layer of water helps to slow-down the evaporation rate of the fluid. The procedure was initially pursued on a clean silicon surface to verify the evaporation phenomenon for the “coffee stain” as shown in Figure 1a. The self-assembly at the water droplet was tested on a silicon nitride surface (see Figure 1b) before deposition on actual SOI magnetometer devices. The green color specifies the nanoparticle deposition area in Figure 1a and the silicon nitride membrane area in Figure 1b, although nanoparticle assembly covers the whole area beyond the green area in Figure 1b. The magnified image of the edge of the silicon nitride membrane shows a clear contrast in Figure 1c. The nanoparticles form a close packed distribution in the self-assembly as shown in Figure 1d. This confirms the formation of a single layer self-assembly of nanoparticles on a silicon nitride surface using the water droplet technique. Multiple self-assembled layers of nanoparticles can also be achieved by increasing the concentration of the prepared nanoparticle solution. The water droplet procedure is explained by schematics (Figure 2a–c) and is extended to describe the assembly on top of magnetometer devices in the sections below.

### 3.2. Schematic of Single Layer Self-Assembly

An additional hydrophobic drop of deionized water is used as an interfacial layer to reduce the “coffee stain” mechanism. This extra water droplet is in between the target silicon surface (or the device) and the nanoparticle solution. A schematic diagram of the process is displayed in Figure 2. A side view of the schematic is shown in Figure 2a, where a drop of ferrofluid was poured on top of the water droplet. The surface tension of the water droplet beneath the nanoparticle solution drives the self-organization of the nanoparticles. The whole set-up was placed inside a closed chamber to further reduce the evaporation rate. The nanoparticle concentration, ferrofluid and water droplet volumes, and environment temperature and pressure conditions were also controlled to best achieve the results. The top view of the schematic, shown in Figure 2b, displays the nanoparticle layer on top of the water droplet on top of the devices. The water droplet covers the whole chip (5×5 mm) and hence it covers the whole pattern of devices. The schematic image of Figure 2 is a representation of a single resonator only for the purpose of clarity.

As the slow evaporation of water occurs [22], the layer of self-assembled nanoparticles on top of the hydrophobic water surface settles and lays down on the silicon surface (Figure 2c). The final bonding forces between the nanoparticles and the silicon surface are strong enough to hold them during nanofabrication processes. The nanoparticle concentration was also best chosen to achieve the single layer of self-assembled nanoparticles. This section explains the deposition procedure for a generic case. The quantities and conditions are explained in Section 4 and Section 5 for the experiments performed.

## 4. Patterning Nanoparticles of the Device Shape (Negative Resist)

The nanoparticles used in [3] were stable single domain nanoparticles (55 nm in size) having a large magnetic moment. It would be interesting to fabricate devices with higher sensitivity that can also measure magnetic nanoparticles with a lower magnetic moment and smaller size. For that purpose, devices were made having higher sensitivity, using HSQ, to form nanoparticle assemblies to the device shape. To demonstrate our device fabrication process, nanomechanical torsional paddles of various square dimensions (2,3, and 4 μm) were created. For each type of paddle size, the length of the torsional rod attached to each side of the paddle varies from 500 nm to 5 μm, and the width was designed to be 150 nm.

For the purpose of using smaller nanoparticles, superparamagnetic magnetic nanoparticles (magnetite, 20 nm in size) were selected for device characterization. The magnetic nanoparticle solution (${\mathrm{Fe}}_{3}O_{4}$ suspension from Sigma-Aldrich, 20 nm in size, 5mg/mL concentration, and 0.865 g/mL density) was diluted to a 1:50 ratio in toluene solvent and mixed using a vortex mixer (VWR-VM-3000 mini vortexer) for 1 min to produce a homogenous solution. The nanoparticles droplet volume of 0.05 μL was placed on top of the water droplet with a micro-pipette. The nanoparticles were coated with oleic acid for a number of reasons including biomedical compatibility, reduced agglomeration (controlled inter-particle distance), surface charge control, and controlled dipole coupling.

**Figure 3:**
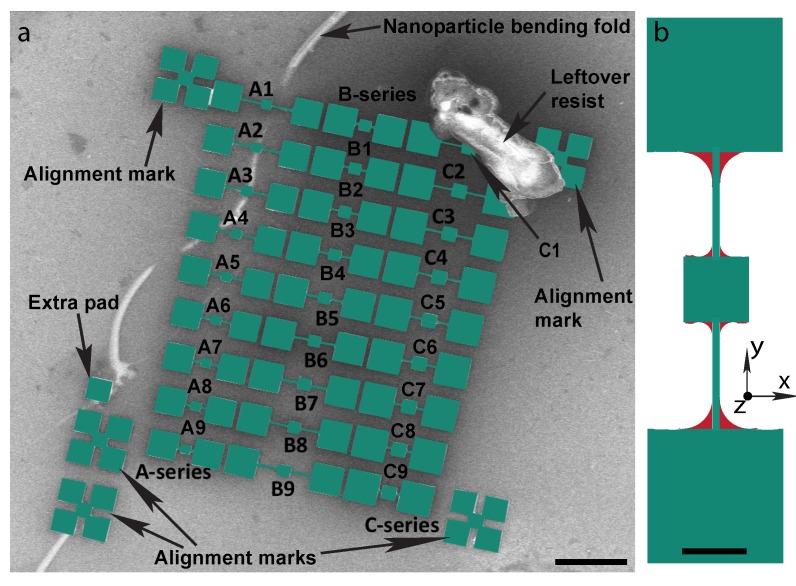
False color SEM image of self-assembly of nanoparticles on SOI fabricated devices. (**a**) Three rows of devices are named as A, B and C series with numbers 1–9. Green color shows the suspended silicon devices with a silicon oxide layer on top of the silicon base (gray color). The alignment marks are the edges of the devices and are also suspended and shown in green color. A single extra pad beside the alignment mark was left behind after etching. The scale bar is 10 μm. (**b**) Schematic of the anchoring of nanoparticles at the corners of the device. The green color is the device and red color indicates the anchored nanoparticles. The scale bar is 3 μm.

The magnetic nanoparticles were deposited on the SOI fabricated devices by using the scheme shown in Figure 2. A set of the devices with nanoparticles deposited is shown in Figure 3. A set of pads of different sizes (smaller and larger than the paddles of the devices) were made beside the alignment marks for confirmation of the released devices. Three pads smaller than the device were etched away, whereas another (larger than the devices, and labeled extra pad in Figure 3a) was still there beside the alignment mark which helped to give the real time etching rate of the silicon oxide layer. The green color represents the single layer self-assembled nanoparticle deposition on top of the devices.

The evaporation of water during the drying procedure in the nanoparticle deposition causes the nanoparticle film to bend and fold locally as seen in Figure 3a. The devices A1–A4, in Figure 3a, were underlying the bending fold of nanoparticles. The devices clear from the bending region were used in the magnetometry measurements. A leftover resist is seen on top of the C1 device in Figure 3a that got stuck during the critical point drying procedure.

Another phenomenon observed, besides the bending of the nanoparticle film, was the anchoring of nanoparticles across the corners of the devices. A schematic is shown in Figure 3b. The nanoparticle film breaks at the edges of the device and torsional rod, and falls on the base silicon, leaving behind the curvature around the corner resulting in anchoring. The formation of this anchoring gives information about the tensile strength and crystallinity of the nanoparticle film. The hexagonal close packed structure helps the formation of anchoring regions. However, this anchoring region is not desired in the experiment, and slows down the resonator and affects its resonance frequency. Therefore, we proceeded further to remove the extra nanoparticle anchors. The devices in the A, B, and C series were compared for torque magnetometry measurements in the sections below.

### 4.1. Cleaning the Anchored Nanoparticles at the Edges of the Devices

The anchored regions affect the clamping points of the torsional rod, and therefore the dissipation mechanism affects the nanomechanical torque amplitude and the quality factor of the device. It was then necessary to remove the extra clamping, provided by the anchoring of the nanoparticles, to reduce the damping. For this purpose, a focused ion beam (FIB) was used for cleaning. A high energy gallium ion (Ga+) beam in FIB was employed to cut and clear the extra nanoparticles attached at the corners of the device.

**Figure 4:**
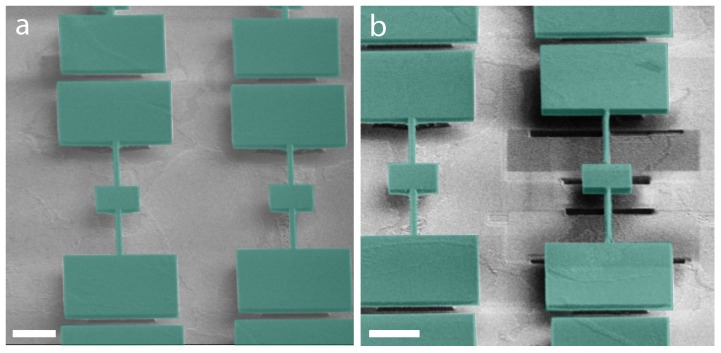
Cleaning nanoparticles from the corners of the device (B9). (**a**) False-color SEM image of two unclean devices: B9 (left) and B8 (right) (**b**) False-color SEM image of the clean device B9 (on the right as the image has been rotated 180° with respect that shown in (**a**)). All scale bars are 3 μm.

**Figure 5:**
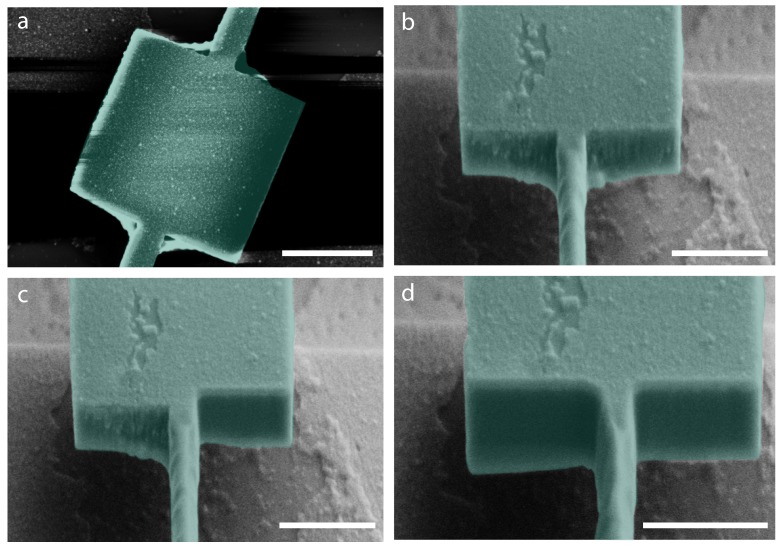
Cleaning steps of the device (B9) using a FIB. (**a**) Anchored unclean device (**b**); side view of partially cleaned device. The torsional rod is cleaned in this step. (**c**) Cleaning one edge by cutting the paddle by 100 nm. (**d**) Cleaned device from both sides. All scale bars are 1.2 μm.

The clamped and anchored devices are shown in Figure 4a. The first device in this series (named B9) was cleaned using FIB (Figure 4b). The torsional rods were rastered with a low dose beam, while the edges of the device paddle and support paddles were cleaned with a high dose to clear up the anchored deposition. The details of the cleaning procedure are shown in Figure 5. The focused ion beam (Zeiss Nvision 40) was utilized in the milling mode (current = 13 nA) for cutting the paddle and in the rastering mode (current = 1 pA) for cleaning the torsional rods.

In the first step, shown in Figure 5b, the torsional rod is cleaned with a beam dose that just rasters the surface and cleans the rods. In the next step, the torsional paddle and the support paddle edges facing the anchoring were cleaned with a high dose to cut through the paddles (Figure 5c). Figure 5d displays a section of the whole cleaned device. Some redeposition of melted material by the FIB underneath the paddle and melting of material on the torsional rod was observed as seen in Figure 5d. This removed the anchoring sections but changed the resonator shape a bit.

**Figure 6:**
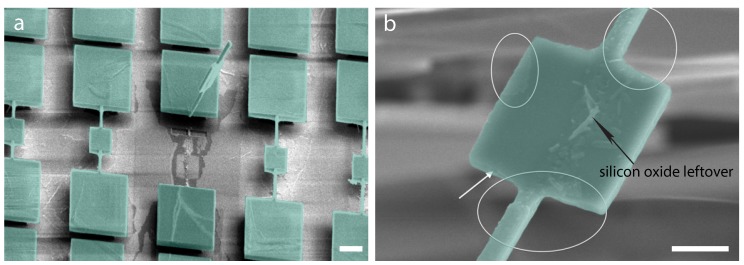
Device (B5) cut to see the nanoparticles on the bottom side. (**a**) False-color SEM image of the cut device. The scale bar is 2.5 μm. (**b**) False-color SEM image of the cut device on the bottom side. The white arrow indicates the oxide layer surface on the bottom side. The encircled areas show a small number of nanoparticles at the edges. The scale bar is 1.2 μm.

During the self-assembly of nanoparticles and after the device cleaning, there might be consequent residual deposition of nanoparticles on the backside of the paddle. It was then necessary to reveal the nanoparticle deposition, whether or not present on the backside of the device. For this purpose, one of the devices (B5) was sacrificed by cutting from one side of the torsional rod and flipping over to see the bottom side of the device, as shown in Figure 6. Figure 6a shows the B5 device cut and flipped over. A magnified image of the cut device is shown in Figure 6b. One can see small portions (encircled) of the nanoparticle deposition that emerged on the bottom side when the nanoparticle film broke during the deposition and rested itself on the paddle. The rest of the contrast is the oxide layer remnants on the bottom side of the paddle.

It is clear from Figure 5 that nanoparticles were deposited on the top side of the resonator, so that the magnetic properties were coming mainly from the single layer square shape self-assembly of nanoparticles. There were 14,285 nanoparticles counted on the top of paddle of device B9. The resultant magnetic properties were the sum of all nanoparticles on the paddle, in a given direction.

### 4.2. Nanomechanical Torque Magnetometry of Nanoparticles on the Devices

**Figure 7:**
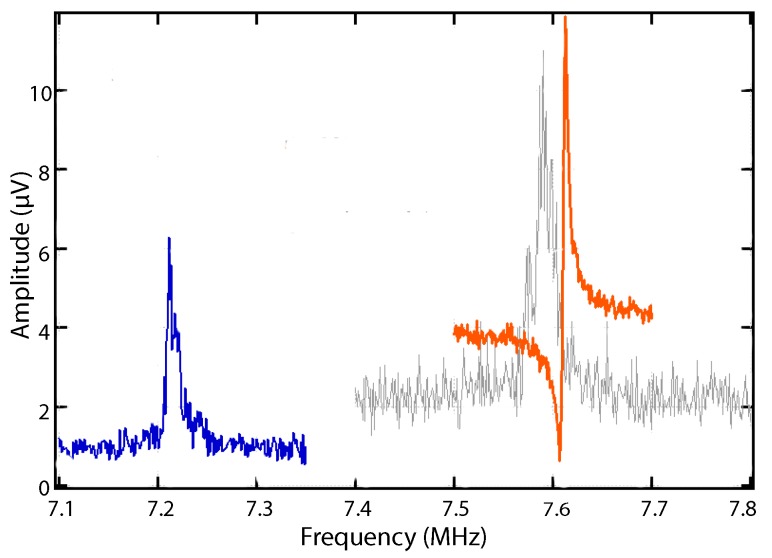
Resonance peaks of the B9 device in vacuum conditions. Gray color shows the thermomechanical peak for the untrimmed device at room temperature. Blue color is the thermomechanical peak for the trimmed device at room temperature. Orange color represents the driven peak of the untrimmed device at 50 mV at 18.25 K.

The cleaning and cutting of the resonator affected the nanomechanical properties of the device. Figure 7 shows the shift in frequency of the trimmed compared to the untrimmed B9 device.

The quality factor of the flexural mode for the device (B9) changes from 18 (untrimmed) to 36 (trimmed) for the thermomechanical peak at room temperature, with a shift of resonance frequency from 7.59 MHz (untrimmed) to 7.22 MHz (trimmed) as shown in Figure 7. With a small reduction in size and anchor-free device, the frequency has reduced to a lower value. There might be a few nanoparticles on the backside of the resonator and clamping points as shown in Figure 6b. The low quality factor is likely due to these additional nanoparticles on the backside of the resonator and clamping points. The cleaning of the nanoparticles was performed on the top surface of the support paddles and torsional rods only.

**Figure 8:**
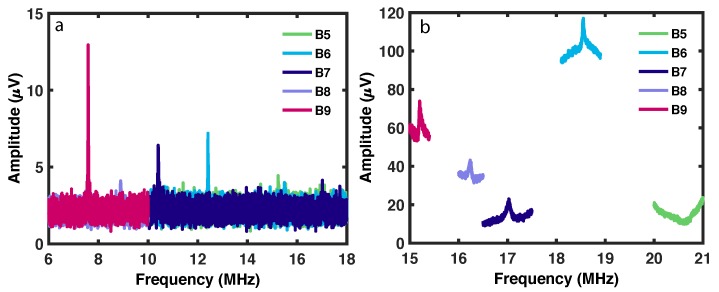
Resonance peaks of the B-series devices in vacuum conditions at room temperature. (**a**) Flexural mode of the devices; (**b**) torsional mode of the devices.

The magnetic torque acted upon the magnetic nanoparticles by the external magnetic fields and the magnetic moment was converted to mechanical torque, such that the magnetic torque was read at the mechanical resonance of the device. The transduced mechanical torque was then optically detected by an interferrometric method. The resonance modes of the devices in the B-series are shown in Figure 8. These resonance modes were measured before cleaning with the FIB. The devices were comparatively in the high frequency range as compared to the nanomechanical devices in the silicon nitride membrane [3]. The increasing length of the torsional rod from device B5 to device B9 changes the flexural and torsional resonance modes to the lower frequency. The flexural and torsional modes were confirmed with the optical raster scan of the resonator. A similar method was used for raster scans and finite element simulations as described in [3]. Device B9 was then selected for further magnetic measurements, particularly as it had a higher amplitude than the other devices.

Magnetic torque and, therefore, magnetic susceptibilities are good measures to characterize the magnetic nanoparticles. Nanomechanical torque magnetometry works on the principle of magnetostatic torque, where an external magnetic field (H) on the magnetic moment (m) exerts torque (τ) , following $\tau =m\times \mu_{0}H$, where $\mu_{0}$ is the permeability of free space. The magnetostatic torque is then transferred to the device and drives a mechanical deflection of the paddle. The mechanical deflection is then detected by a displacement transduction method of free-space interferometry.

The interferometry set-up for detection consists of a helium neon laser (632 nm) aligned with optics on an optical bench to the vacuum chamber ($10^{-5}$ bar), where the device chip is placed. The laser beam is aligned and focused on the paddle edge for flexural and torsional mode detection. The detected signal is the interference output of the reflected light from the paddle surface and from the silicon base layer of the substrate. The applied field on the device comes from a permanent magnet on a linear rail. The external field axis (*x*-axis) was chosen perpendicular to the torsional rods, and another AC coil was placed under the chip to provide $H_{AC}$ along the *z*-axis. The product of these quantities produced torque in the *y*-axis. The reference signal was provided to the coil by a high frequency lock-in amplifier (Zurich Instruments), which also detected the interferometric signal on a photodiode. More details of the detection mechanism have been explained in [3,23,24]. The measured resonance modes are representing the deflection of the paddle because the laser spot was focused on the paddle in the detection scheme. The support pads are fixed and do not have nanoparticles on them. The nanoparticles on support pads were cleaned with a focused ion beam during the cleaning process.

**Figure 9:**
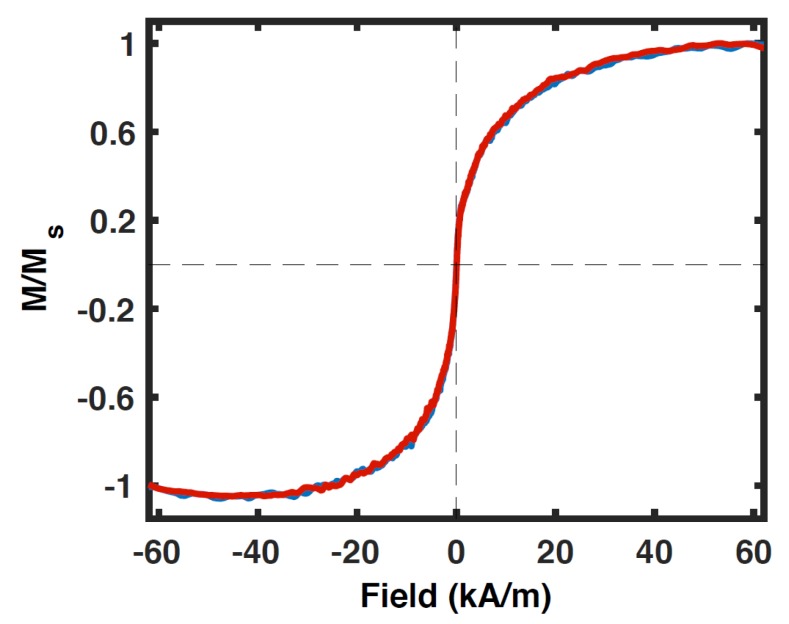
Magnetic hysteresis of superparamagnetic nanoparticles (20 nm) at room temperature under vacuum conditions. The normalized torque is plotted versus $H_{x}^{\mathrm{DC}}$ field. Blue color represents the high to low field sweep, whereas red color is the low to high field sweep.

Magnetic hysteresis of magnetite nanoparticles (20 nm) was obtained at room temperature and at low temperature. The magnetic hysteresis was obtained at the fixed resonance frequency of the resonator (torsional resonance frequency). Figure 9 shows the hysteresis curve of the superparamagnetic nanoparticles at room temperature. It can be clearly seen that nanoparticles are superparamagnetic as the hysteresis loop is closed, passes through the origin and has a steep slope near the origin (indicating high magnetic susceptibility).

## 5. Patterning Nanoparticles of Any Shape (Positive Resist)

**Figure 10:**
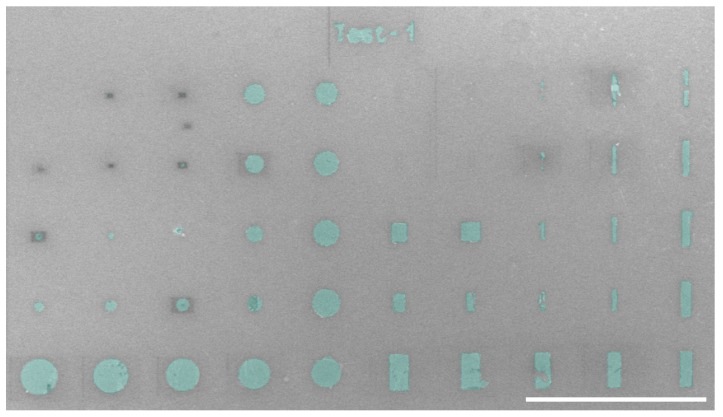
Nanoparticles self-assembly using PMMA. False color SEM image of patterned circles and rectangles of magnetic nanoparticles. The scale bar is 50 μm.

For the self-assembled nanoparticles on the device, shape anisotropy can be introduced into the system, by changing the shape of the individual nanoparticles and/or by depositing the nanoparticles in a specified shape. To control the magnetic susceptibilities, the nanoparticles were deposited in geometrical shapes as shown in Figure 10. It shows the nanoparticle deposition on a silicon surface in different shapes and sizes.

### 5.1. Preliminary Tests of Patterning Nanoparticles on a Silicon Surface

The process producing the self-assembly of nanoparticles on the nanomechanical devices requires the use of various chemicals in the nanofabrication, such as poly methyl methacrylate (PMMA), acetone, pentane, hydrogen silsesquioxane (HSQ), and α-methylstyrene (ZEP). In order that the intended nanoparticles (${\mathrm{Fe}}_{3}O_{4}$, 20 nm in size, in toluene solvent, from Sigma-Aldrich) were first tested and deposited in combination with these chemicals on the silicon surface, Section 4 has details of devices made using HSQ, whereas the following subsections will describe the results of the nanoparticles compatibility with the PMMA, ZEP and acetone. Our goal in the current study is the assembly of nanoparticles on the resonators and performing torque magnetometry, not the study of chemical interactions.

To investigate the patterning behavior of nanoparticles using PMMA, a raith design was created for the experiment, to be used in electron beam lithography (EBL). Various shapes including rectangles, squares, and circles were created in the initial raith design. The raith design was used in the EBL for patterning the shapes on the resist. The deposition of nanoparticles after the EBL exposure was performed at later stages of nanofabrication.

The process started with cleaning the silicon-on-insulator (SOI) chips (5×5 mm in lateral size) by the piranha process in nanofabrication. The SOI chips have a top silicon layer of 300 nm or 145 nm, the buried oxide layer thickness is 1 μm, and the base silicon layer is ~700 μm. The chips were then washed with deionized water and blow dried with a nitrogen air flow. The clean chips were then spin coated with PMMA, in a double layer structure.

Firstly, the poly-methyl methacrylate (PMMA 950 K A8 from Microchem Corp., now Kayaku Advanced Materials, Inc., Westborough, MA, USA) was spin-coated on the clean chips using a Laurell spinner with an initial speed of 500 rpm for 5 s ramped up to 4000 rpm for 40 s. The coated chips were baked on the hot plate (Laurell hot plate) at 180 ${}^{\circ }$C for 10 min and cooled down for 10 min. A second layer of the poly-methyl methacrylate (PMMA 495 K A2 from Microchem Corp., now Kayaku Advanced Materials, Inc., Westborough, MA, USA) was spin-coated on top of the PMMA 950 K layer using the same speeds of the spin coater. The second PMMA layer was baked following the same steps as for the first layer. The double layer spin coating of PMMA is usually undertaken to avoid the extra backscattering of electrons from the EBL exposure.

The coated chips with PMMA were then carried to the EBL for electron beam exposure with 10 keV for an optimum exposure and for reduced effect of charging. The resist was first exposed on the alignment marks in the raith design for the beam alignment. A dose test was carried out separately to choose the appropriate area dose (200 $\mu C/{\mathrm{cm}}^{2}$). The electron beam exposed various shapes and sizes of circles, rectangles, and squares. The exposed resist was developed in isopropyl alcohol (IPA) for 80 s and development was stopped by cleaning the chips with deionized water for 90 s.

The nanoparticle solution was deposited on top of the developed resist using the water droplet during the deposition process explained in Section 3.2. As the water evaporates and the solution dries out, the nanoparticles get into the holes created by the EBL exposure in the resist. The nanoparticles lay down on the bare silicon surface inside the shaped holes. The rest of the area of the chip is still covered by the resist. The resist lift-off was carried out, to remove it completely, in the acetone for 24 h. The idea to avoid using other chemicals was to reduce the exposure of nanoparticles to new chemicals, thus avoiding any chemical changes, and to avoid high temperature because it can affect the magnetic properties of nanoparticles.

Figure 10 shows an SEM image of the circles and rectangles of the deposited assemblies of nanoparticles. The deposition size varied between 20 μm to 100 nm in diameter for the circles and 20 μm to 100 nm in length for the rectangles. The figure shows the array of well-defined and precisely aligned various shapes of the magnetic nanoparticle assemblies. The nanoparticles also followed the pattern of the text “Test-1” as the title of the trial experiment.

**Figure 11:**
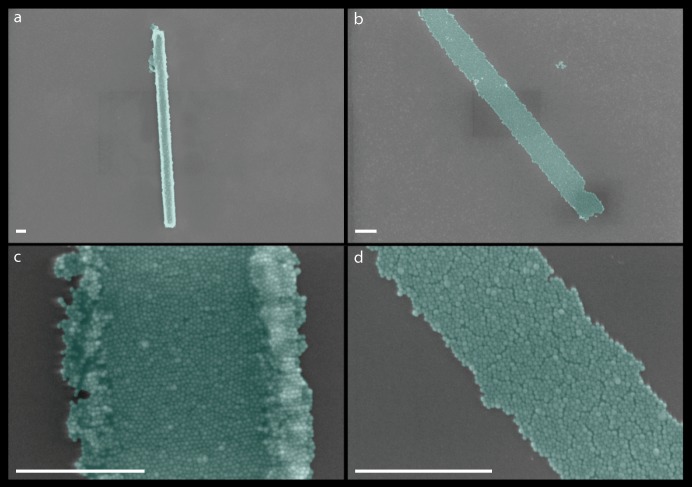
Clearing the borderline of nanoparticles assembly. (**a**–**d**), False color SEM images of patterned rectangles of magnetic nanoparticles. Panel (**c**) is the magnified image of an area in panel (**a**). Panel (**d**) is the magnified image of an area in panel (**b**). All scale bars are 500 nm.

A cluttered borderline was formed when a double layer of PMMA was spun onto a chip as shown in Figure 11a,c. The overexposure of the resist caused the nanoparticles to congregate at the edges of the assembly. The problem was resolved by removing one layer of the PMMA, that is, only using a single layer of the PMMA. Therefore, the two-layer approach that we initially used, following the procedure of Zhu Diao [7], did not work in our case. The clear edges and boundaries of the nanoparticle assembly can be seen in Figure 11b,d. Several dry-runs were performed to achieve the desired results using PMMA-950 K A8, PMMA-495 K A8 and PMMA-495 K A2 (depending upon the molecular weight and the viscosity). This process of shape formation was much easier than the stencil mask process [25], and resulted in high resolution, high contrast, and sharp edges and corners [7].

**Figure 12:**
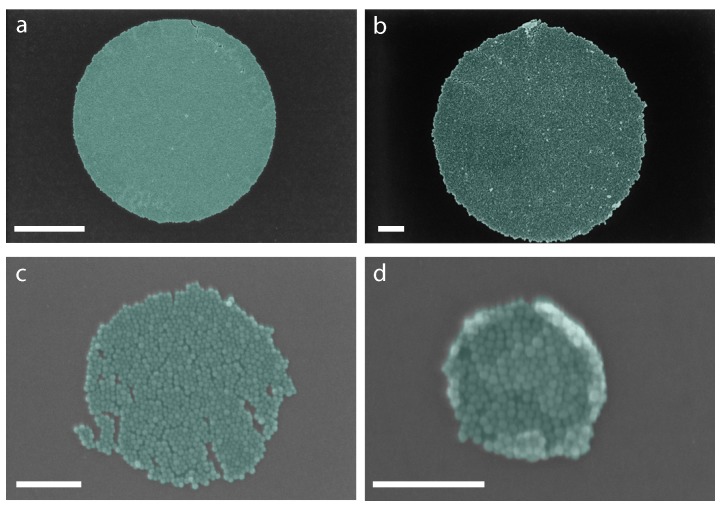
Nanoparticle circles by mask fabrication. Scale bar for (**a**) is 2 μm. Scale bar for (**b**–**d**) is 200 nm.

Circles of various sizes were also deposited on the silicon surface as shown in Figure 12 in a magnified view. It can be seen that nanoparticles, in a circular shape, can be deposited down to 200 nm diameter in size with ease.

### 5.2. Patterning Nanoparticles in Geometrical Shapes on SOI Devices

**Figure 13:**
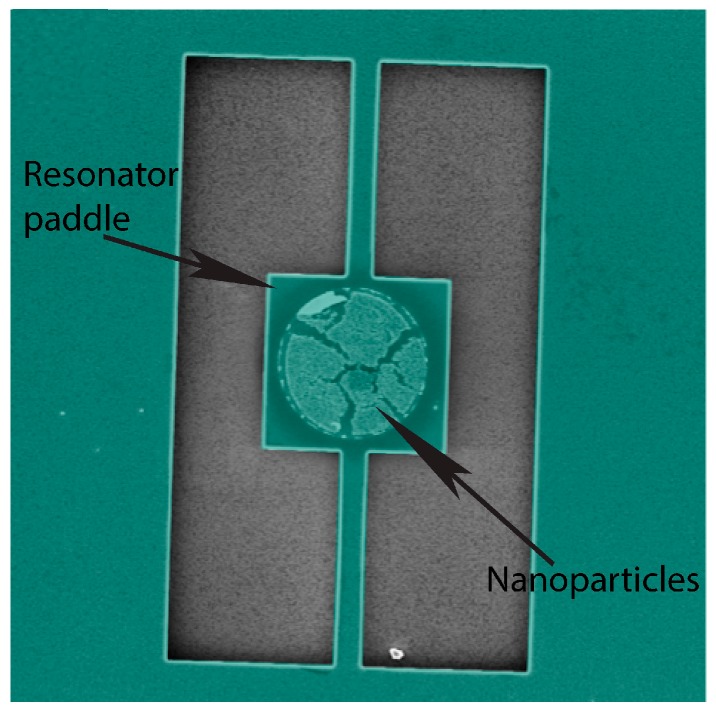
Nanoparticle circles on SOI by nanofabrication. The resonator paddle is 6 μm wide.

After the successful geometrical deposition on the silicon surface using PMMA, the magnetic nanoparticles were then deposited on the devices, in circular shapes shown in Figure 13. These devices were made using a positive α-methylstyrene (ZEP) resist. The dark circle around the nanoparticles on the devices is due to the backscattering of electrons in the poly methyl methacrylate (PMMA) resist layer during the electron beam lithography (EBL). The full procedure of device fabrication is explained in the Supplementary Materials. The magnetic nanoparticles were deposited by the method described in Section 3.2.

### 5.3. Resonance Modes of the Devices

**Figure 14:**
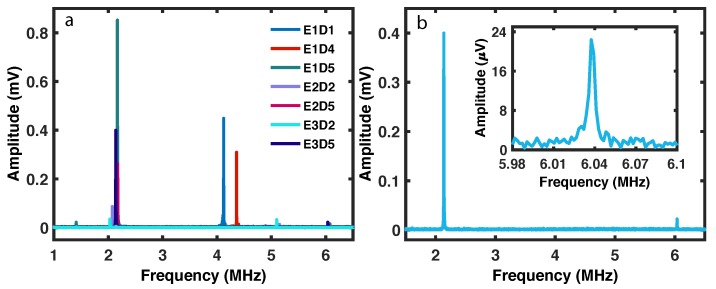
Resonance spectra of nanoparticle circles on SOI devices under vacuum conditions at room temperature. (**a**) Torque amplitude of various devices with nanoparticles. (**b**) Resonance spectrum of an individual device. Inset shows the Lorentzian shape of the resonance curve for the higher frequency peak.

The devices were then measured for the mechanical signal in frequency sweeps of the lock-in amplifier. Figure 14 shows the measured torque amplitude of various devices (named in the legend). The flexural modes of the devices were around 2 MHz while the torsional modes were at higher frequency from 4 to 6 MHz. The amplitude in Figure 14a shows that these devices had a much better signal than the device cleaned in the previous section. The resonance modes of an individual device is shown in Figure 14b. The displacement, torque, and magnetic moment sensitivities were $7.5\times 10^{-14}m{\left({\mathrm{Hz}}^{1/2}\right)}^{-1}$, $7.0\times 10^{-20}\mathrm{Nm}{\left({\mathrm{Hz}}^{1/2}\right)}^{-1}$, and $5\times 10^{7}\mu_{B}$, respectively, for the device shown in Figure 14. These values are calculated by the standard method explained in [7,23,26].

The SOI nanomechanical devices described in the present paper have higher sensitivity than the silicon nitride nanomechanical devices published earlier ($7\times 10^{8}\mu_{B}$) [3]. Therefore, better device fabrication resulted in measuring a smaller magnetic moment. The properties of current SOI nanomechanical devices, shown in Figure 7, Figure 8 and Figure 9 and Figure 14, were measured in vacuum conditions at room temperature (apart from the low temperature curve in Figure 7).

In future, to measure the magnetic properties of small-scale self-assemblies of magnetic nanoparticles in ambient conditions, one needs to fabricate highly sensitive devices that have better magnetic moment sensitivity in ambient conditions than the current magnetic moment sensitivity under vacuum conditions. The simple geometry of nanoparticles assembly and better sensitivity of such devices may lead to understanding the magnetic interactions of small-scale magnetic assemblies under ambient conditions. This motivation has already led to the fabrication and optimization of nanophotonic optomechanical split-beam nanocavity resonators, useful for ambient condition magnetic characterizations (magnetization, susceptibility, and spin resonances). These laboratory-on-chip devices, made for ambient conditions, are presented in [23].

## 6. Conclusions

In summary, the nanoparticle deposition in geometrical shapes on top of nanomechanical devices has been presented. The processes involved standard SOI-based nanofabrication and liquid interface nanoparticle deposition. Various geometrical shapes of small-size nanoparticle deposition have been demonstrated, i.e., square, rectangular, linear, and circular. The devices with nanoparticles on them were characterized initially by finding the flexural and torsional resonance frequencies. The extra nanoparticles on the torsional rod and support paddle were cleaned using a focused ion beam and finally the magnetic characterization was performed. The magnetic hysteresis of deposited nanoparticles has been demonstrated for the proof of concept of a small-scale single layer self-assembly of nanoparticles on the magnetometers. The presented hysteresis is a collective property of a given number of nanoparticles. The quantification of such a small number of nanoparticles has never been performed before using torque magnetometry. It is a completely novel achievement of our work. This process can be extended in future to only a few nanoparticles, or even a single nanoparticle, on an ultrahigh sensitivity magnetometer. Finally, the nanoparticle assembly procedures are not limited to the ferrimagnetic magnetite nanoparticles described in this paper, as they should work for other types of nanoparticles in a stable suspension.

## Supplementary Materials

The following are available online at https://www.mdpi.com/1422-0067/21/3/984/s1.

## Acronyms

BOE	Buffered Oxide Etch
CPD	Critical Point Drying
EBL	Electron Beam Lithography
HF	Hydrofluoric Acid
HSQ	Hydrogen Silsesquioxane
ICPRIE	Inductively Coupled Plasma Reactive Ion Etching
NMP	N-Methyl-2-Pyrrolidone
PMMA	Poly Methyl Methacrylate
SOI	Silicon-on-Insulator
ZEP	α−Methylstyrene
