# Supplementary material for: Assembling Magnetic Nanoparticles on Nanomechanical Resonators for Torque Magnetometry
Source: Int J Mol Sci. 2020 Feb 2;21(3):984. doi: 10.3390/ijms21030984 (PMC7037736; doi:10.3390/ijms21030984)
Supplement: Supplementary file 1 [file ijms-21-00984-s001.zip › ijms-660004-SI.pdf]

International Journal of
Molecular Sciences

MDPI

Article

Supplementary Materials: Assembling Magnetic Nanoparticles on Nanomechanical Resonators for Torque Magnetometry

Tayyaba Firdous [1,2], and David K. Potter [1,*]

[1] Department of Physics, University of Alberta, Edmonton, AB T6G 2E1, Canada
[2] National Research Council of Canada, 11421 Saskatchewan Dr NW, Edmonton, AB T6G 2M9, Canada
* Correspondence: dkpotter@ualberta.ca

Received: 19 November 2019; Accepted: 22 January 2020; Published: 2 February 2020

The nanomechanical devices were fabricated in the silicon-on-insulator (SOI) chips. In the following sections, the general procedures followed for the device fabrications are presented. Some special cases are also described for better explanation of the related experimental problems.

S1. Difficulties in Fabrication of Nanomechanical Devices in Silicon Nitride Membranes

The design of nanomechanical triple paddle devices was created in raith software and then implemented in the FIB machine (Zeiss NVision 40). If the ion beam conditions are not chosen wisely for the material to be fabricated, strange shapes of the device can occur after the ion beam exposure. One example of a deformed device, with a paddle curling up, is shown in Figure S1. Figure S1 shows the bending of the membrane itself around the device edges, which could cause a change in the resonance frequency. The quality of the device degrades accordingly. The maximum distortion can be seen in the torsional rod that has been curled into a spring like structure. The ion beam dose was 80 pA and the accelerating voltage was 30 kV with a stage tilt of 54° for the fabricated device in Figure S1. The stage was tilted such that the ion beam was perpendicular to the device surface. A Zeiss NVision FIB machine can simultaneously acquire the SEM images during the milling process. The SEM column was at an angle of 36° from the device surface.

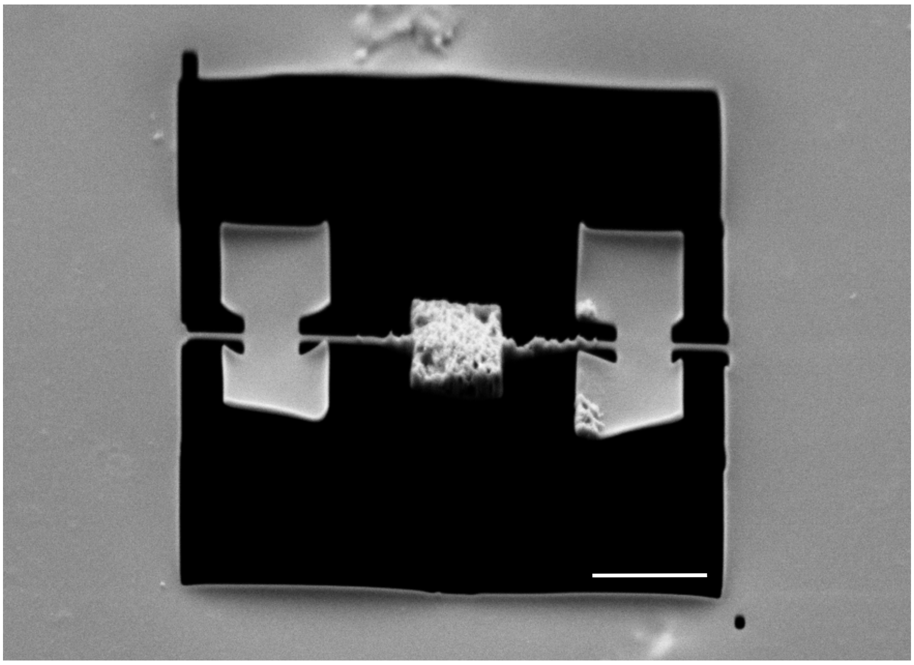

Figure S1. Curling of device. Triple paddle device fabricated in the silicon nitride membrane. The scale bar is 2 μm.

S2. Fabrication of Nanomechanical Devices in SOI Chips

The fabrication process was started on the silicon-on-insulator (SOI) chips (5 × 5 mm in lateral size). The chips were diced from a 6 inch diameter wafer with a top silicon layer of 300 nm or 145 nm, a buried oxide layer thickness of 1 μm and a base silicon layer of about 700 μm. The process recipe has structured steps, some of which are explained below. Steps 1–9 are named as the first cycle, and 10–15 are named as the second cycle.

For negative resist devices, only steps 1-9 below in the first cycle are required and then nanoparticles are deposited by the method in Section **??**. For positive resist devices, the first cycle is used for device fabrication and the second cycle is used for magnetic nanoparticle deposition. In the first cycle, the process is very similar for negative and positive resists, except that the specific parameters are listed below.

The fabrication process includes the following steps in the first cycle:

1. Raith design
2. Piranha cleaning
3. Resist spin coating
4. Electron beam lithography (EBL)
5. Cold development
6. Plasma etching (ICPRIE)
7. Resist lift-off
8. Oxide etching (BOE) to release the devices
9. Critical point drying (CPD)

Raith design: The raith design was created for both resist types (positive (ZEP) and negative(HSQ)). The HSQ (hydrogen silsesquioxane, XR-1541 from Dow Corning, MI, USA) and ZEP (11% methyl styrene and chloromethyl acrylate copolymer and 89% anisole solvent, molecular weight of 57,000, ZEP520A from ZEON Corp., Tokyo, Japan) were used at the NanoFab, University of Alberta.

Piranha cleaning: The first step is the standard cleaning procedure with a combination of sulfuric acid (H_2SO_4) and hydrogen peroxide (H_2O_2), which removes the organic compounds on the substrates.

Standard operating procedures were followed for piranha cleaning. The sulfuric acid and hydrogen peroxide were mixed in a ratio of 3:1. The chips were placed vertically in a honeycomb teflon tray and covered with a teflon cover. The assembly was then immersed in the piranha solution for 20 minutes in glass containers. The chips were then rinsed with water five times and then air blowed for drying.

Resist spin coating: The resist was spin coated for a thickness of 150 nm. The chips were dried at 180°C on a hotplate (Laurell hotplate) for 5 minutes and then cooled down on paper towels for 5 minutes. The resist was allowed to be at room temperature from the fridge temperature for proper spinning. The chip was placed on top of the spin coater (Laurell spinner) and a few drops of resist were dropped on top of the chip so that whole chip was covered with resist. The spinner was then ramped up to 100 rpm for 5 s, and then was spun at 4000 rpm for 40 s. After spin coating the desired number of chips, the chips were baked at 180°C for 10 minutes (150°C for 5 minutes for HSQ), and then were allowed to cool on clean paper towels with aluminum foil cover in order to avoid radiation exposure to the resist before EBL. The chips were transferred to the gel box to carry them to the EBL room. The whole procedure was performed with clean metal tweezers. The negative resist (6% HSQ) was diluted in a MIBK solution in a ratio of 1:2 (2.5 ml of HSQ: 5 ml of MIBK), the positive resist (ZEP) can be diluted in anisole (1:1) to get a standard spinning curve because it evaporates over time.

Electron beam lithography (EBL): EBL (Raith 150-Two machine) transfers the design pattern for the first cycle of raith software on the positive or negative resist used. The chips were loaded on the sample stage of the EBL and the vacuum was turned on. The sample stage was aligned in the machine. After the stage alignment, a standard manual procedure was used for the electron beam focusing and pattern writing. A pre-dose test was carried out for proper dose values. An area dose of 80-125 pC/cm^2 for ZEP (200-400 μC/cm^2 for HSQ) was used in various resists, since the calibration curve for each resist will change over time. The sample was unloaded after the design writing with an electron beam on the resist. For a positive resist, the electron beam exposes the areas of the device, and for a negative resist, the electron beam exposes the inverted areas of the device. An electron voltage of 30 kV for ZEP, 10 kV for HSQ and aperture of 10 μm diameter was used in the EBL. An average beam current was 37 pA for ZEP and 22 pA for HSQ.

Cold development: The ZEP resist was developed in a cold environment for better spatial resolution and sharp edges. 15 ml of ZED-N50 and 15 ml of IPA were cooled at -15°C on a Stir-Kool SK-12D cold plate. Silicone oil was used to provide optimal contact of the beaker with the cold plate for heat transfer, and a small magnetic stirrer (60 rpm) was used in each glass beaker for temperature homogeneity. A standard procedure was followed to setup the cold plate. The chips were immersed in the ZED-N50 solution for 20 s, then in IPA for 20 s, and then air dried with nitrogen purge afterwards. The HSQ was developed in 25% tetramethylammonium hydroxide (TMAH) for 60 s.

Plasma etching (ICPRIE): Reactive ion etching (RIE) was used for etching the silicon layer under the exposed resist. The inductively coupled plasma RIE (ICPRIE) was used in an Oxford Instruments PlasmaLab System. The chamber was conditioned with a dummy silicon wafer for 10 minutes, with standard conditions (16 sccm of SF_6,12 sccm of C_4F_8, RF power of 25 W and a ICP power of 3,500 W). The chips were then loaded into the chamber with silicone oil at the bottom. The chips were exposed to the plasma etching for 30 s for 145 nm, and 40 s for 300 nm. The chips were cleaned from the bottom side with IPA to remove the oil, after the completion of the etching process.

Resist lift-off: The resist lift-off was carried out in heated NMP (N-Methyl-2-Pyrrolidone) at 70°C for 30 minutes. The chips were placed in IPA for 2 minutes and then air dried. The resist lift-off can also be performed in acetone for 24 hours.

Oxide etching - BOE: The silicon oxide layer is the buffer layer, where undercut was created to release the devices. The oxide layer etching is also called buffered oxide etching (BOE). A concentrated solution in a 10:1 ratio of hydrofluoric acid (HF) and ammonium bifluoride was used in teflon containers (because it will eat the glass containers). HF etches at 55 nm/min. The chips were placed in the HF solution for 32 minutes (paddle size was 3 μm = 3000 nm, so 1500/55 = 27 min etch and we added 5 more mins), and then placed in deionized water for 5 minutes. The chips were then transferred

to an IPA solution from water. A standard procedure was followed for cleaning the wet deck after HF use with $CaCl_2$. The etching time varies with the device dimension; for a bigger paddle it takes a longer time to etch.

Critical point drying (CPD): The chips were then transferred to the critical point dryer for drying to avoid stiction. The whole transfer procedure was performed in the IPA solution medium so that the chips remained in a liquid environment until drying in a critical point dryer. The temperature in the critical point dryer reaches up to 0°C and chips remain in it for 1 hr in a CO_2 environment. The released and dried devices from the critical point dryer were saved for further use. The drying procedure in pentane was also performed for some of the chips separately.

The second fabrication cycle includes the following steps:

10. Resist spin coating
11. Electron beam lithography (EBL)
12. Resist development
13. Magnetic element deposition
14. Resist lift-off
15. Critical point drying (CPD)

Resist spin coating for second cycle: A second layer of resist poly methyl methacrylate (PMMA) was used for coating in the spinner. A bilayer resist was created with PMMA 495 K A2 and PMMA 950 K A8 using the same recipe as described in step 3. The chips were first placed in the acetone to provide a liquid environment to the released devices. The first resist was dropped on top of acetone on the chip (allow 10 s for resist to take place of acetone) when placed in the spin coater. The resist was then spun and baked at the described temperature. The resist is supposed to flow in the undercut below the device. After baking the first resist layer, the second resist layer was spin coated and then baked at a given temperature (180°C for 10 minutes, and cooled for 5 min).

Electron beam lithography for second cycle: EBL for electron beam exposure was done at 10 kV electron voltage and at an area dose of 200 $\mu C/cm^2$. A triple point alignment in EBL was carried out before the exposure for each write-field area. The steps in sequence are: load in EBL, adjust (10 kV and 10 aperture), do alignment on the diamond cut and measure current, adjust XY and UV coordinates (absolute), go to first alignment mark (AM), do the write-field (WF) alignment, go to the AM of the WF, center to the AM, go to 3-point alignment, adjust UVW to local, adjust P1 ($10\mu m, 10\mu m$) three times, flag place in center, position to next AM, read position using dropper and click check, adjust three times, adjust P1 ($10\mu m, 10\mu m$), P2 ($90\mu m, 10\mu m$), and P3 ($10\mu m, 90\mu m$), add design in position list, adjust position ($50\mu m, 50\mu m$), scan layer 63 (AM only), do it three times, set optimum dose, scan for layer 0, reset UV for each WF.

Resist development for second cycle: The PMMA resist was developed in a solution of MIBK:IPA (1:3) for 60 s and then in IPA for 20 s. The chips were washed with deionized water and air dried later.

Magnetic element deposition: The magnetic element deposition was done at this stage. The magnetic nanoparticles were deposited as described in Section **??**.

Resist lift-off: For magnetic nanoparticles, the chips were placed in acetone for 24 hours for resist lift-off.

Critical point drying: To dry the chips at the final step, chips were placed in the IPA and transferred to the critical point dryer. The chips were examined under the SEM for their final pattern and were then ready to use for magnetometry measurements.

© 2020 by the authors. Licensee MDPI, Basel, Switzerland. This article is an open access article distributed under the terms and conditions of the Creative Commons Attribution (CC BY) license (http://creativecommons.org/licenses/by/4.0/).
