# Supplementary figures and images for: Assembling Magnetic Nanoparticles on Nanomechanical Resonators for Torque Magnetometry
Source: Int J Mol Sci. 2020 Feb 2;21(3):984. doi: 10.3390/ijms21030984 (PMC7037736; doi:10.3390/ijms21030984)

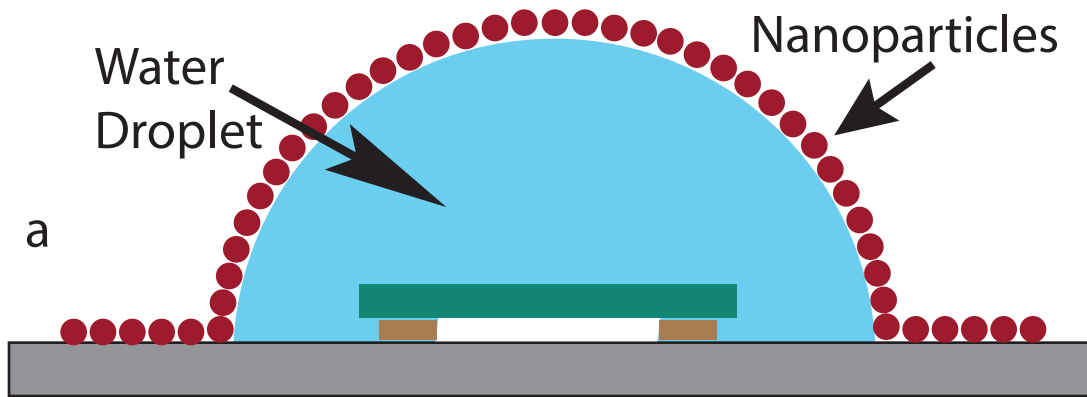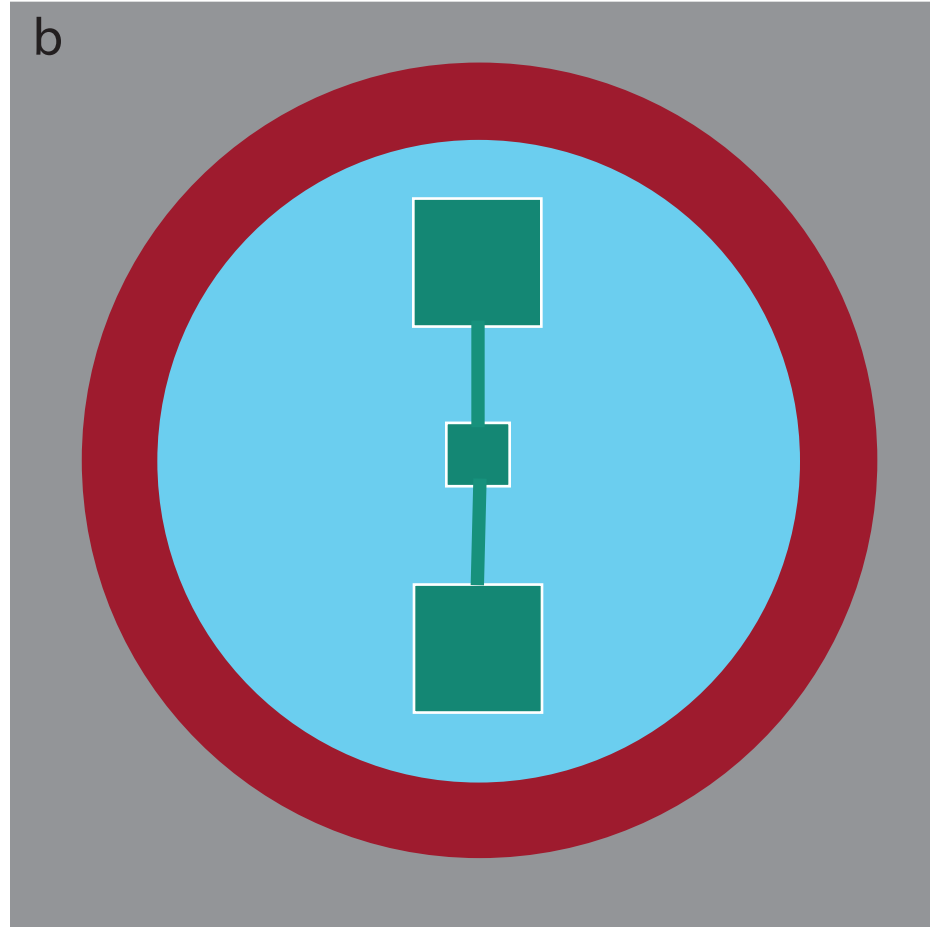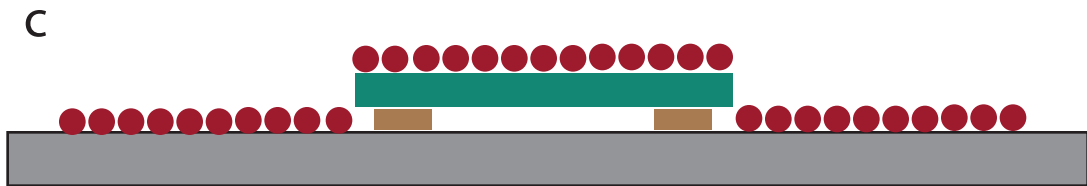

Supplement: Supplementary file 1 [file ijms-21-00984-s001.zip › Definitions/ch5-fig1-schematic.pdf]

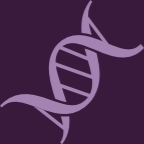

# International Journal of *Molecular Sciences*

Supplement: Supplementary file 1 [file ijms-21-00984-s001.zip › Definitions/ijms-logo-eps-converted-to.pdf]

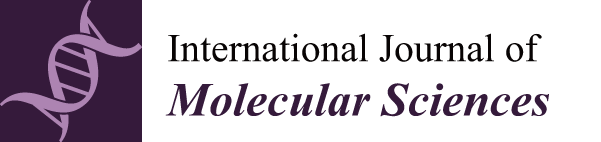

Supplement: Supplementary file 1 [file ijms-21-00984-s001.zip › Definitions/ijms-logo.png]

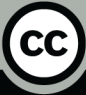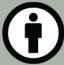

BY

Supplement: Supplementary file 1 [file ijms-21-00984-s001.zip › Definitions/logo-ccby-eps-converted-to.pdf]

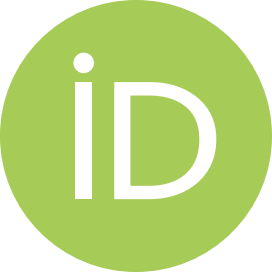

Supplement: Supplementary file 1 [file ijms-21-00984-s001.zip › Definitions/logo-orcid-eps-converted-to.pdf]

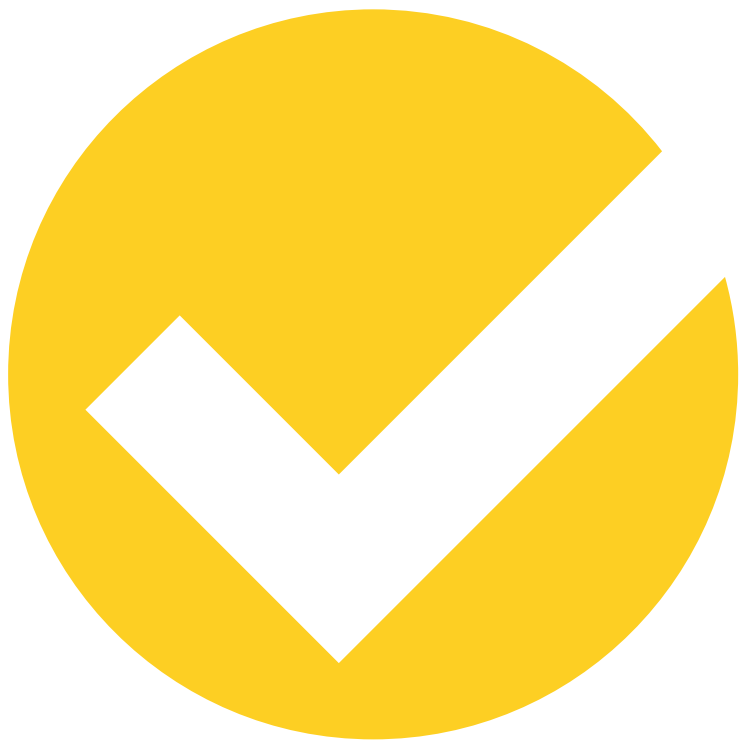

check for  
updates

Supplement: Supplementary file 1 [file ijms-21-00984-s001.zip › Definitions/logo-updates.pdf]

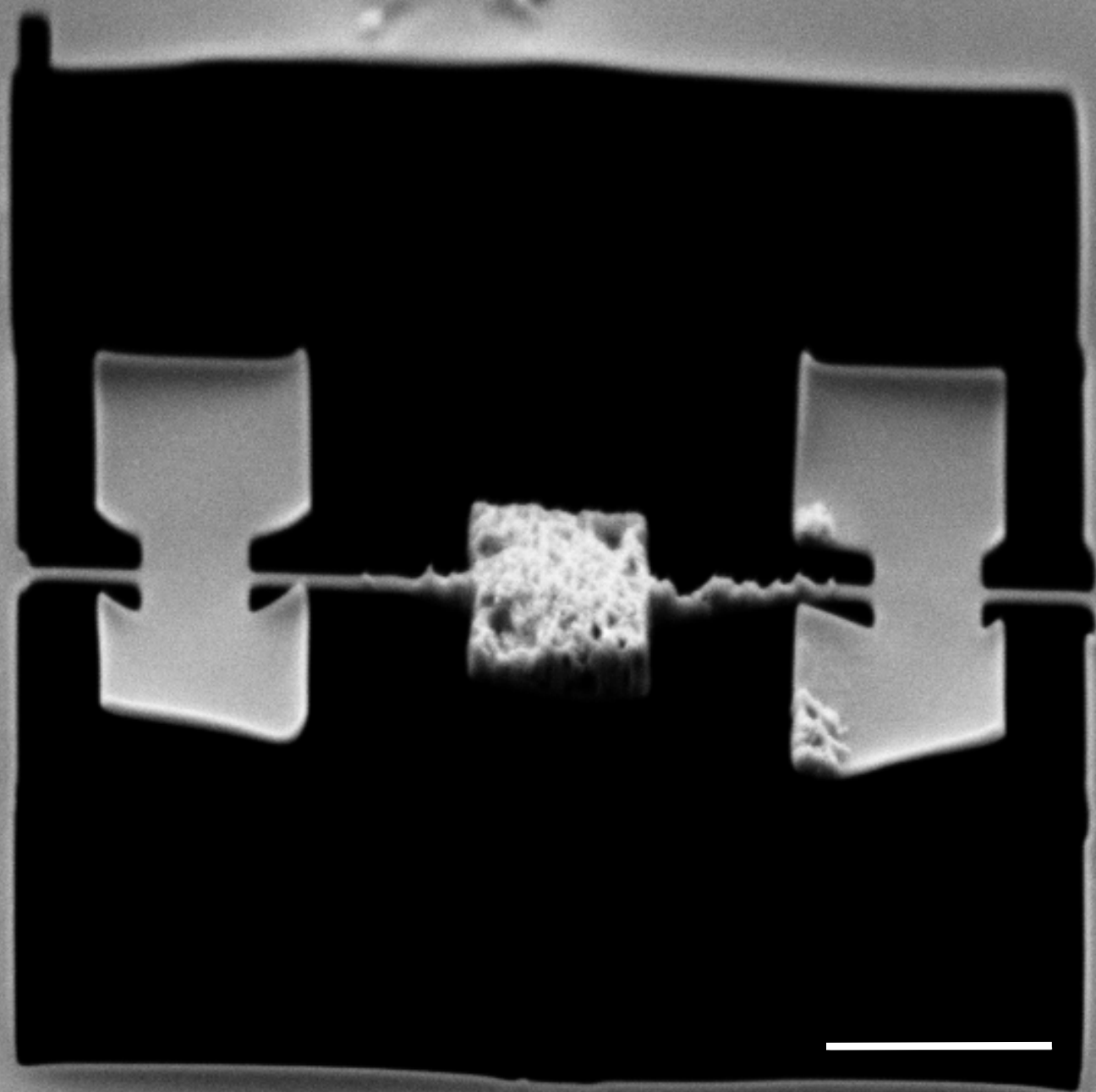

Supplement: Supplementary file 1 [file ijms-21-00984-s001.zip › Figure-S1.pdf]
